# Photochemical valorization of hydrogen sulfide: a study of UV-induced decomposition pathways

**DOI:** 10.1039/d5ra04250j

**Published:** 2025-10-07

**Authors:** Hassnain Abbas Khan, Ali Elkhazraji, Mohammad Abou-Daher, Damian P. San Roman Alerigi, Khalid Hazazi, Aamir Farooq

**Affiliations:** a Clean Energy Research Platform, Physical Sciences and Engineering Division, King Abdullah University of Science and Technology (KAUST) Thuwal 23955-6900 Kingdom of Saudi Arabia aamir.farooq@kaust.edu.sa; b Department of Mechanical Engineering, King Fahd University of Petroleum & Minerals Dhahran 31261 Saudi Arabia; c Interdisciplinary Research Center for Hydrogen Technologies and Carbon Management, King Fahd University of Petroleum & Minerals Dhahran 31261 Saudi Arabia; d Exploration and Petroleum Engineering Center-Advanced Research Center (EXPEC ARC), Saudi Aramco Dhahran 34465 Saudi Arabia

## Abstract

Hydrogen sulfide (H_2_S) is a toxic and environmentally hazardous gas, yet it also represents a potential source of valuable hydrogen. This study investigates the direct gas-phase decomposition of H_2_S into hydrogen (H_2_) and elemental sulfur (S_*x*_) using UV-C light sources through both photolytic and photocatalytic pathways. Experiments were conducted using a 220 nm UV laser and a 254 nm mercury (Hg) lamp in distinct reactor configurations. Photolysis of 5% H_2_S/N_2_ achieved conversion efficiencies of up to 44% and 52% within 60 minutes using the laser and Hg lamp, respectively. In flow experiments (space velocity ∼15 h^−1^), conversion decreased to 13–16%. In both static and flow modes, sulfur deposition on optical surfaces hindered UV transmission, thereby reducing overall efficiency. Incorporating a CuS photocatalyst significantly enhanced H_2_S decomposition, reaching 66% conversion under UV-C illumination. X-ray photoelectron spectroscopy (XPS) confirmed the presence of mixed Cu^+^/Cu^1+δ^ valence states in CuS, enabling localized surface plasmon resonance (LSPR) that promotes charge separation and catalytic activity. These findings underscore the promise of UV-C-driven H_2_S splitting as a sustainable approach for hydrogen and sulfur co-production, offering a cleaner alternative to conventional treatment methods.

## Abstract 2

Hydrogen sulfide (H_2_S) is a toxic and environmentally hazardous gas, yet it also represents a potential source of valuable hydrogen.

## Graphical abstract

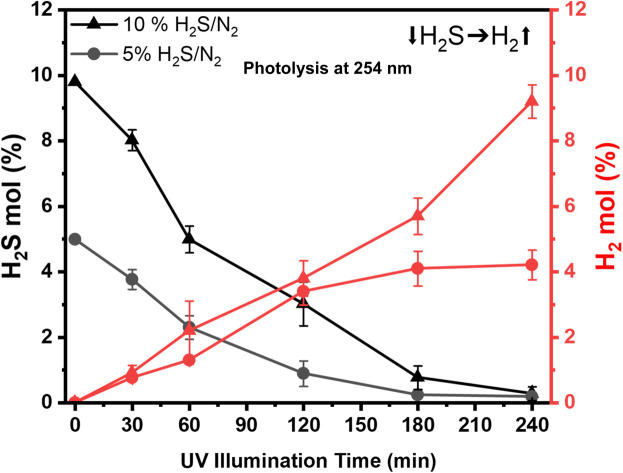

## 1. Introduction

Hydrogen sulfide (H_2_S) is recognized as a noxious gas that poses significant environmental and health risks due to its toxic nature; for example, its odor threshold ranges between 0.4 ppb and 1.5 ppm.^1^ Exposure to concentrations as low as 100 ppm is highly toxic and can lead to death within 48 hours, while levels exceeding 700 ppm are fatal within minutes.^2^ H_2_S is naturally abundant in crude oil and natural gas well sites.^3^ Its oxidation can form sulfur oxides, which pose significant health and environmental risks and cause severe corrosion.^4^ Downstream, stringent treatments (pre-/post-combustion) are necessary in refineries and power plants to control the emissions of sulfuric pollutants (*i.e.*, H_2_S, SO_*x*_).^5,6^

Various methods are currently used to capture and eliminate H_2_S. The most common techniques include solubilization in an aqueous basic media^7^ and the Claus process.^8^ The latter is widely utilized in industry due to its economic viability and established operation framework. It involves a two-step reaction.^8^ Initially, H_2_S undergoes thermal oxidation above 1000 °C to produce sulfur dioxide (SO_2_) and water at elevated temperatures. Then, a catalytic step employing activated aluminum(iii) or titanium(iv) oxides converts the remaining H_2_S and SO_2_ to elemental sulfur and water. The process can achieve a conversion efficiency of up to 98%, leaving about 2–5% of H_2_S unconverted as tail gas.^9–11^ This residual H_2_S poses environmental and safety concerns, as it is a toxic and malodorous gas.^12^ Hence, additional purification steps are necessary to remove this residual H_2_S,^13,14^ including catalytic oxidation or absorption processes, which add complexity and cost to the overall operation.^15,16^ Furthermore, the presence of contaminants in the feed gas, such as aromatic compounds (*e.g.*, benzene, toluene, and xylenes) can affect the reaction kinetics^17^ and lead to catalyst deactivation and the formation of soot, which clogs the catalytic reactors and reduces their efficiency,^18^ From the perspective of energy intensity, the need for high temperatures to facilitate the reaction also contributes to increased energy consumption and operational costs.^19,20^ These limitations pose significant challenges that demand the development of novel alternatives for H_2_S decomposition that are energy-efficient and sustainable.

Considering these limitations, researchers have explored alternative methods for H_2_S treatment and removal^21–23^ Recent research endeavors have focused on harnessing H_2_S as a source of hydrogen (H_2_).^23,24^ Such a paradigm shift would eliminate H_2_S while positioning it as a contributor to the hydrogen economy. Several methods of producing hydrogen from H_2_S have been described in the literature, including thermal,^25^ electrochemical,^26^ plasma-chemical,^27^ and photocatalytic.^28–32^ However, many of these methods suffer from the overoxidation of elemental sulfur, leading to the formation of toxic sulfite and sulfate-bearing compounds. Light-driven methods are of particular interest because of the abundance of sunlight and the continuous improvement in the efficiency of light-emitting diodes. Several studies have studied the photo-physics of the H–S bond under the UV, visible, and IR spectra^12,33,34^ to uncover light's potential as a driving force for H_2_S decomposition. However, the gas-phase splitting of H_2_S to produce hydrogen and elemental sulfur and the nature and yields of these reaction products have not been explored in depth. From a practical perspective, the design of reactors for gas-phase photolysis and photocatalysis is critical for optimizing contact time and light exposure.

Solar light offers significant advantages as an energy source for several reasons. One key benefit is that photon energy can be transferred directly to the molecules without any external power input; the only requirement is a transparent matrix at the excitation wavelength.^35^ Additionally, advanced technologies enable the filtering of the solar light spectrum to isolate specific wavelengths, such as UV, visible, or IR, tailored to particular applications. This capability enhances the versatility and efficiency of solar energy utilization. Recent studies have explored the photodecomposition of H_2_S into hydrogen gas in the liquid phase using semiconductor and noble metal co-catalysts under UV/vis light irradiation.^36^ The combination of UV-C (200–280 nm) and VUV (<200 nm, typically 172 nm from Xe excimer lamps) radiation has been shown to significantly enhance the degradation rates of H_2_S, suggesting that advanced oxidation processes (AOPs) can be effectively employed for this purpose.^37,38^ Some studies have utilized air as an intermediary to decompose H_2_S, though achieving net-zero sulfur remains elusive with this approach.^39^ These studies often utilized low concentrations of H_2_S, resulting in the formation of sulfur-containing acids that can cause photocatalyst deactivation. While non-oxidative gas-phase H_2_S splitting has received limited attention, it holds great promise for the simultaneous production of hydrogen and elemental sulfur.^21,40^ The non-oxidative photochemical approach shows minimal to no interference of other gases, such as CO_2_, CH_4_, and N_2_, present in natural gas streams.^40^

This study investigates the efficiency of direct gas-phase H_2_S splitting under UV-C irradiation, both photolytically and photocatalytically. Specifically, the study explores two UV-C wavelengths (220 nm and 254 nm) due to the high absorption cross-section of H_2_S at 220 nm and the more significant solar flux at longer (254 nm) wavelengths. Instead of directly using solar radiation, this work utilizes a laser source at 220 nm and a lamp at 254 nm. Applying a light source at 220 nm, seldom investigated in the literature, is a significant contribution of this research. The results of this work will help advance the technology of photochemical conversion of H_2_S to H_2_ and S.

## 2. Experimental details

The current study uses two distinct UV sources and reactor systems: a Ti:sapphire laser system coupled to a metallic photoreactor, and a Mercury lamp embedded in a quartz photoreactor. The concentration of H_2_S ranges from 1% to 10%, and the decomposition behavior is investigated from ambient temperature (25 °C) to elevated temperature (125 °C) at a pressure of ∼1.5 bar. The H_2_S gas cylinders with purity levels (1–10% in H_2_S nitrogen) are sourced from Gulf Gas Corporation Saudi Arabia. The feed gases H_2_S/N_2_ flowed through the reactor, and the effluents were introduced into a Thermo Fisher scientific gas chromatograph (GC) equipped with two TCD detectors and an Rtx-1 and Al_2_O_3_–Na_2_SO_4_ double-column separation system for the reactivity measurements. A calibration curve was applied to quantify the H_2_S and H_2_ signals. The condensation of sulfur powder could also be observed visually during the decomposition and recorded with a camera. The sulfur is analyzed with X-ray diffraction to identify the phase-type and purity.

### 2.1. Ti:sapphire laser system coupled to a metallic photoreactor

**Fig. 1:**
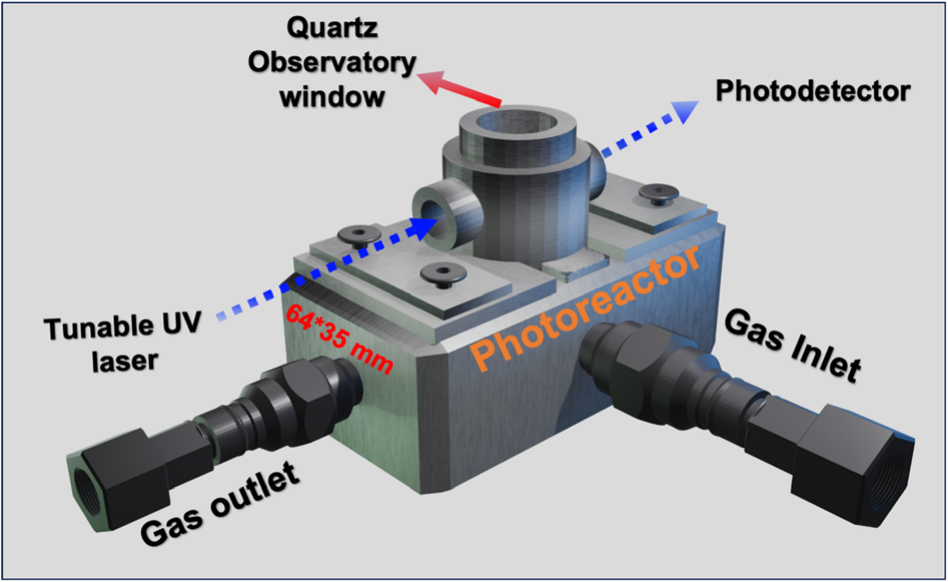
Schematic of the photolysis setup using a modified Harrick photoreactor.

The Praying Mantis™ high-temperature (Harrick Scientific Products) reactor (20 ml volume) with a modified dome is used in this study. The reactor has three UV-grade optical windows for laser illumination with 99.5% UV transmission efficiency. Titanium-doped sapphire (Ti:sapphire) crystal generates the desired wavelength. Ti:sapphire is a solid-state laser medium capable of tunable operation over a broad range of near-infrared (IR) wavelengths. Here, we used a mode-locked laser (Tsunami®), which gave tunable output over 690 nm to 1080 nm. The laser was operated at a repetition rate of 80 MHz and a pulse width of 2 ps. The Ti:sapphire laser was pumped by a 532 nm Nd:YAG diode-pumped solid-state laser (Millennia®). The near-IR photons were upconverted to UV through two consecutive second harmonic generation processes, equivalent to fourth harmonic generation (FHG). Fig. 1 shows a schematic of the optical setup used to test when the laser is used as a laser source coupled with a metallic photoreactor.

### 2.2. Mercury lamp coupled to a quartz photoreactor

**Fig. 2:**
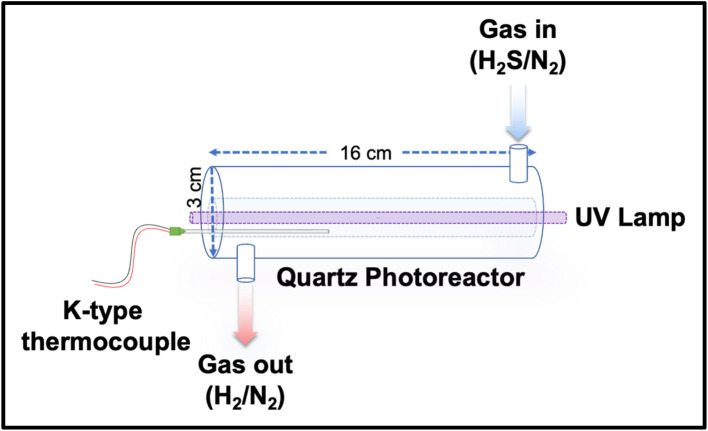
Hollow jacketed quartz photoreactor for H_2_S photolysis.

This study used a double-tube UV-grade quartz reactor and UV lamp. The reactors geometry is shown in Fig. 2. The H_2_S flows through 20 ml volume outer tube. The UV source, a mercury (Hg) lamp (*Ushio G8T5*), is located inside the inner tube and illuminates the reactor directly. This lamp emits a quasi-monochromatic light at 254 nm with an output power of 2.5 W.

## 3. Results and discussion

### 3.1. Photolysis of H_2_S at 220 nm

**Fig. 3:**
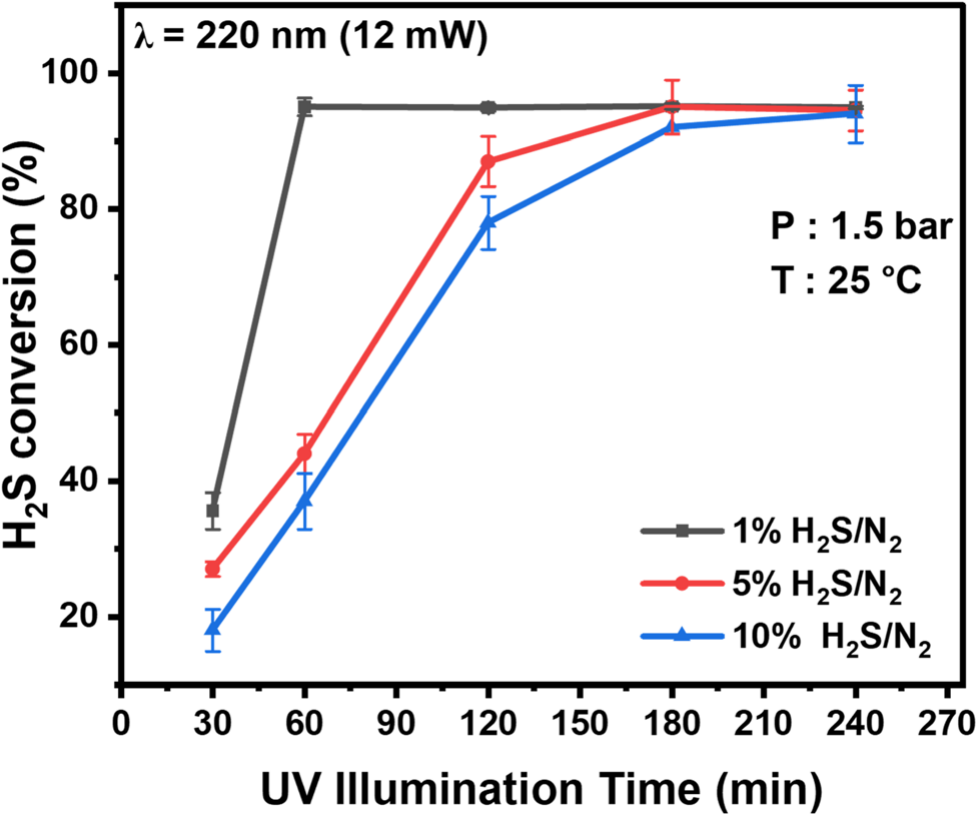
Photolysis of H_2_S at 220 nm in the metallic photoreactor for different H_2_S/N_2_ mixtures as a function of reaction (UV illumination) time.

Fig. 3 shows the photodecomposition of H_2_S as a function of reaction time at a wavelength of 220 nm and an optical power of 12 mW. These experiments were carried out in a batch mode using the metallic photoreactor with three mixtures of varying H_2_S concentrations. These data clearly show the increasing conversion of H_2_S as a function of illumination (or reaction) time. Conversions of 35%, 27%, and 18% are recorded for the 1%, 5%, and 10% H_2_S mixtures, respectively, after 30 minutes of reaction time. These results indicate the rapid initiation of the photodecomposition process. As the light exposure time progresses, the conversion of H_2_S steadily increases, reaching almost 94%, 44%, and 36% at 60 minutes and rising to 96%, 89%, and 76% at 120 minutes for the 1%, 5%, and 10% H_2_S mixtures, respectively. Nearly complete removal of H_2_S is observed after 180 minutes for the 5% H_2_S mixtures, while more than 90% conversion is observed for the 10% H_2_S mixture.

The plots in Fig. 3 confirm the conversion rates follow an exponential decay, typical of first-order reactions, with a rate constant inversely proportional to the initial concentration. The cause of this reduced reactivity is likely due to the condensation of sulfur on the optical windows, which inhibits the transmission of light inside the cavity. This effect was confirmed using a photodetector placed downstream of the reactor to quantify the transmitted laser intensity, which was found to decline with the thickening of the sulfur layer forming on the optical windows, as shown in Fig. S1 (SI). The condensation of sulfur powder produced from photolyzed H_2_S was recorded using an HD camera (see Fig. S2). Sulfur powder condensation was observed on the optical windows soon after the laser beam was propagated through the reactor. As shown in Fig. S2, the initial frame at time 0 reveals clear optical windows. Within only 2 seconds, the distinct appearance of sulfur becomes evident on the optical windows. As the reaction progresses, the sulfur layer thickens and eventually covers substantial portions of the optical windows. The time-lapse recording provides an insightful visual representation of the rapidity with which the reaction proceeds, thereby highlighting the swift onset of sulfur formation. The emergence of sulfur indicates the complete dissociation of H_2_S molecules to hydrogen and sulfur (rather than SH + H formation). We have provided a video recording as SI for visualizing the instant H_2_S decomposition and sulfur formation.

**Fig. 4:**
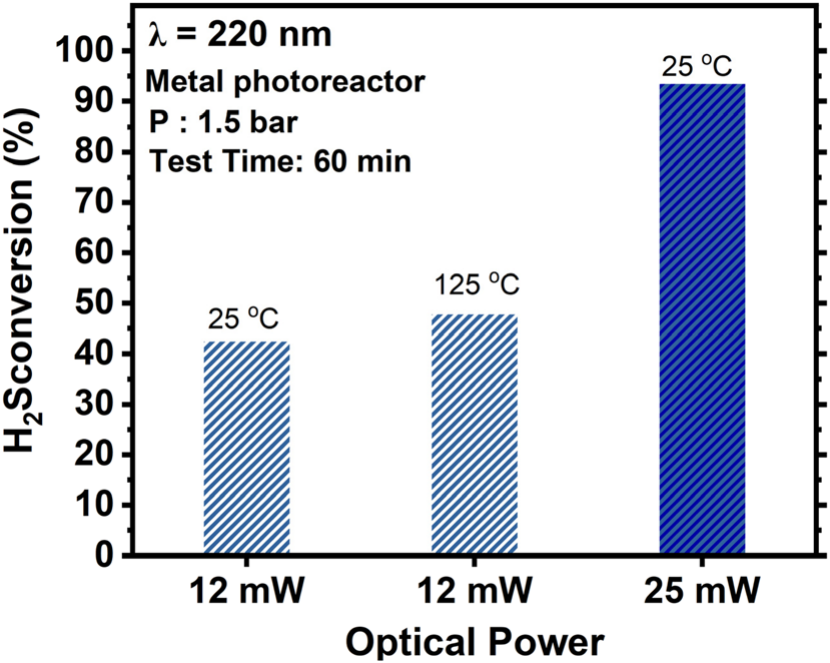
Effect of laser power and reaction temperature on the photolysis of 5% H_2_S at 220 nm.

Fig. 4 shows the impact of optical power on the efficiency of the photodecomposition process. The remarkable 89% conversion achieved with 25 mW of laser power at 220 nm wavelength during a 60-minute reaction is noteworthy. Increasing the laser power from 12 mW to 25 mW resulted in more than 2× increase in H_2_S conversion, indicating a non-linear effect of laser power on the conversion efficiency. Fig. 4 also shows the effect of reaction temperature on H_2_S conversion. Increasing the temperature from 22 °C to 125 °C raised conversion from 42% to 49%. These findings emphasize the interplay between laser wavelength, optical power, temperature, reactor design and solid sulfur accretion, all of which governing H_2_S photodecomposition efficiency. The accumulation of sulfur significantly disrupts this process by obstructing light reaching the H_2_S molecules, scattering or attenuating incident photons.

### 3.2. Photolysis of H_2_S at 254 nm

**Fig. 5:**
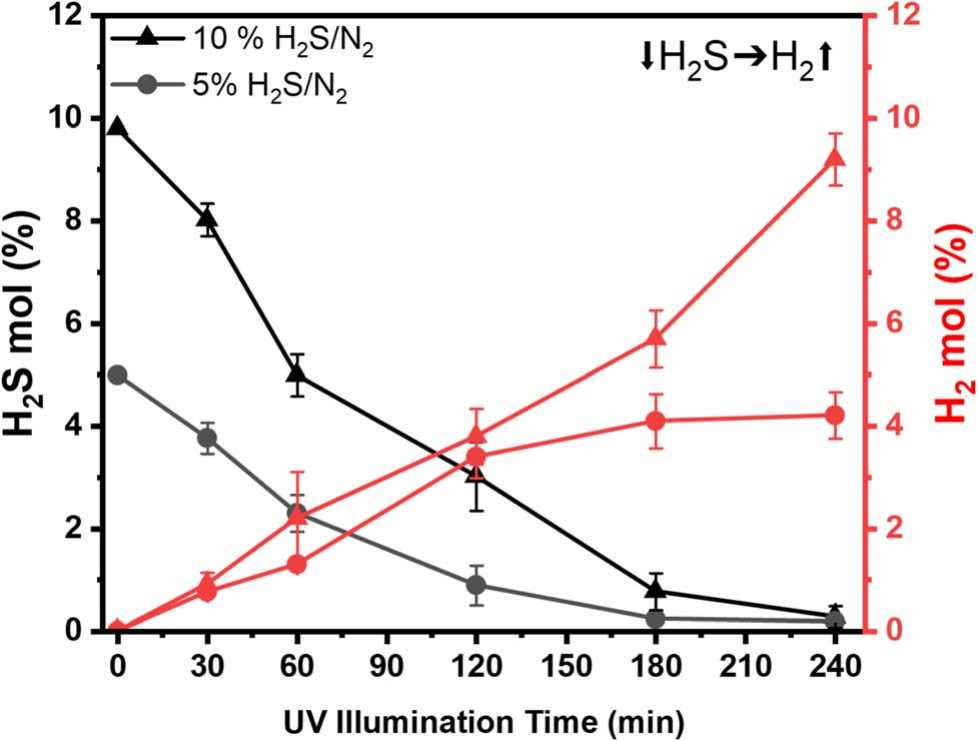
Photolysis of two different mixtures of H_2_S/N_2_ in the quartz reactor using an Hg lamp emitting UV light at 254 nm.

This section discusses the results of H_2_S photodecomposition in the quartz reactor exposed to an Hg lamp emitting UV light at ∼254 nm. The input electrical power of the UV lamp is 8 W and it emits 2.5 W optical power (data provided by Ushio Inc). Fig. 5 shows that gas mixtures containing 5% and 10% H_2_S result in nearly complete H_2_S conversions while yielding stoichiometric amounts of H_2_ moles. Similar to the metal photoreactor experiments, sulfur condensed on the quartz reactor walls (see Fig. S3). Although the reactor was not externally heated in these experiments, the temperature inside the quartz reactor reached ∼50 °C in 3 hours due to the heat dissipation from the Hg lamp.

The observed conversions at specific time intervals plotted in Fig. 5 reveal the progressive decomposition of H_2_S under the influence of UV irradiation. For the 10% H_2_S case, an initial conversion of 19.8% is measured after 30 minutes, reaching 51% after 60 minutes and 69% after 120 minutes. By 180 minutes, the conversion reaches nearly 97%, with the remaining H_2_S mole fraction being 0.27%. This result indicates the continued efficiency of the UV lamp in driving the dissociation of H_2_S over time. The decomposition rate is higher for the 5% H_2_S case than for the 10% H_2_S case. The initial conversion at 30 minutes is 24%, and the conversion reaches 82% in 120 minutes. The quartz reactor becomes entirely covered with sulfur powder (see Fig. S3), confirming the H_2_S decomposition process in 60 minutes. This change in transparency due to sulfur accumulation before and after the reaction is a tangible indication of effective H_2_S conversion under the UV light of the Hg lamp.

On the other hand, the reduced transparency results in lesser UV light transmission, which can explain the change in reaction rate and efficiency of the process. As a result, at later reaction times, the reaction rate is slower than at earlier illumination times. This observation highlights the need to develop sulfur removal and quenching techniques to improve the efficiency of the process.

### 3.3. Conversion efficiency

**Fig. 6:**
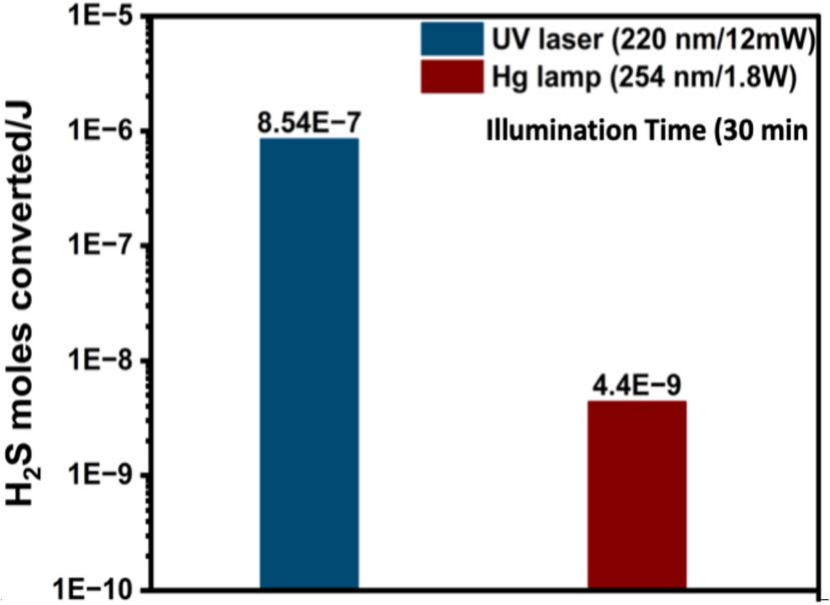
H_2_S conversion efficiency per joule of optical power at 220 nm and 254 nm.

The efficiency of the photolytic process, in terms of moles of H_2_S converted per joule of optical energy, is calculated as follows:
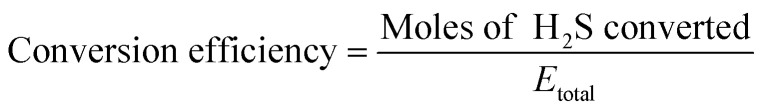


Fig. 6 presents an approach to evaluate the efficiency of photolysis process as the conversion of H_2_S moles per joule of optical power. The calculations are based on effective optical power (based on the optical access area of the reactor). The photolysis conversion efficiency, based on moles of H_2_S converted per joule of optical energy, was 8.54 × 10^−7^ at 220 nm and 4.4 × 10^−9^ at 254 nm, highlighting the superior performance at 220 nm.

**Fig. 7:**
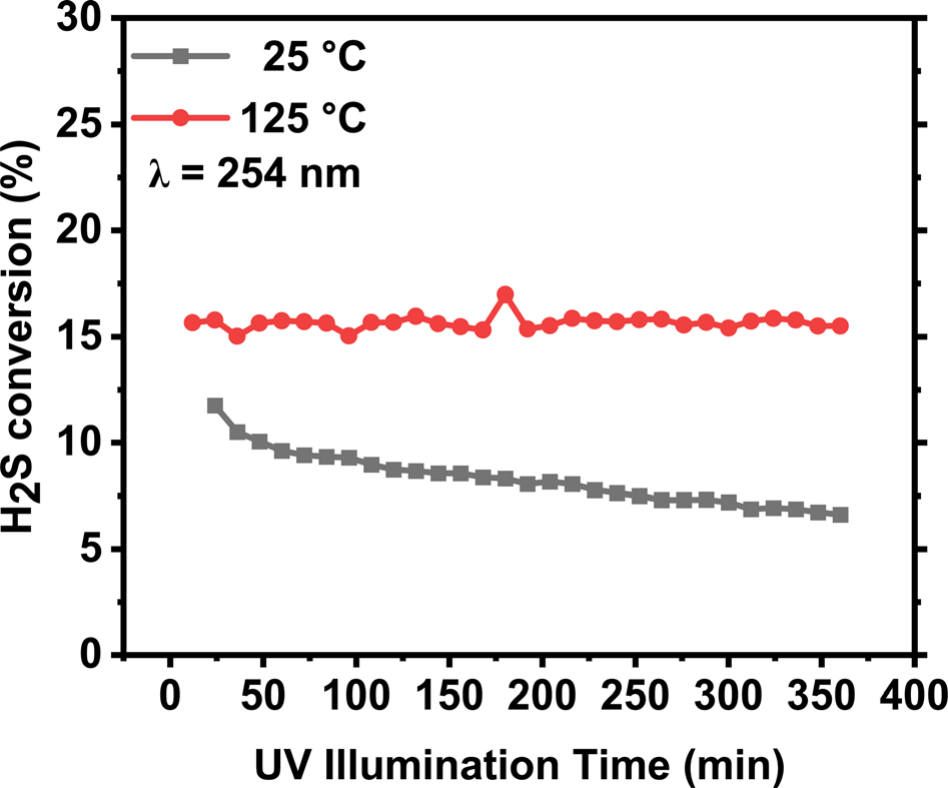
H_2_S conversion in flow experiments in a quartz reactor using an Hg lamp at a GHSV (gas hourly space velocity) = 15 h^−1^ at 25 °C and 125 °C.

Fig. 7 shows H_2_S conversion in continuous flow experiments in the quartz reactor. The procedure adopted here is batch-to-flow transition. The 5% H_2_S mixture is filled in the reactor, and the Hg lamp is illuminated to initiate the reaction. A continuous flow (5 ml min^−1^) of 5% H_2_S mixture is initiated by controlling the outlet valve, resulting in a residence time of 240 s. Gas samples are collected every 12 min and analyzed using an online GC. The flow reactions were carried out over 350 minutes at room temperature (25 °C) and heated (125 °C) conditions. The reactor was heated using a heating tap, and the temperature of the reactor surface was measured using a K-type thermocouple. It is observed that the activity under the room temperature condition experienced a conversion reduction from ∼13% to ∼7%. Meanwhile, under the heated condition (125 °C), the activity remained stable throughout the experiment. At room temperature, the photoreactor became nearly opaque due to sulfur deposition after a 60-minute continuous reaction, significantly reducing UV light transmission and resulting in slower H_2_S conversion. Fig. S4 shows pictures of the reactor after the reaction. In the heated experiments, sulfur was observed only in the colder edges of the reactor during and after the reaction. This observation suggests that sulfur started to melt at 125 °C and condensed in the colder parts of the reactor.

### 3.4. Photocatalytic H_2_S decomposition

**Fig. 8:**
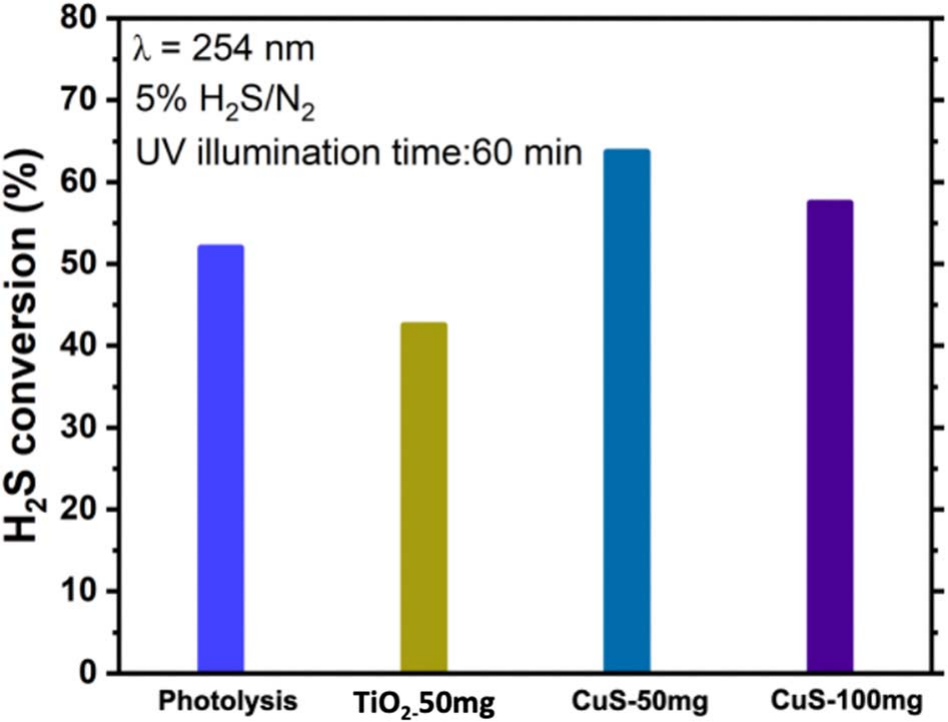
Comparison of photolytic and photocatalytic batch experiments conducted in the quartz reactor with an Hg lamp.

To understand the role of common catalysts in H_2_S conversion, two catalysts, namely commercial TiO_2_ (CristalACTiV™ G5 supplied by Tronox, anatase phase) and an in-house synthesized CuS, were loaded into the quartz reactor by making stable suspension. Details about the synthesis method and catalyst characterizations are given in the SI. The performance of TiO_2_ and CuS is assessed at various loadings and tested over 40–60 minutes in the quartz reactor. Fig. 8 plots the conversion rate for the photolysis and photocatalysis experiments. The results show that the TiO_2_ catalyst did not improve H_2_S decomposition compared to the photolytic reaction. It is important to note that TiO_2_ started to turn yellowish as soon as the reaction began. It is speculated that the formed sulfur resulted in TiO_2_ poisoning. In the case of CuS, a higher rate of reaction was achieved compared to the photolytic reaction, likely due to the plasmonic effect. However, as the quantity of the CuS catalyst in the reactor was increased to 100 mg *via* multiple coatings, the activity slightly decreased due to the reduced UV light transmission. This result suggests that the effective transmission of UV light is crucial for H_2_S photolytic and photocatalytic decomposition.

Table S1 (SI) benchmarks our results against relevant literature on H_2_S photolysis and photocatalysis. Previous gas-phase studies by Baldovi *et al.* (2017),^40^ achieved ∼40% conversion at 254 nm without a catalyst, while TiO_2_-based systems suffered from sulfur poisoning and showed limited efficiencies of 30–52% under UV-C illumination.^39,41^ Aqueous-phase CuS photocatalysis demonstrated ∼58% conversion under visible light,^28^ but direct gas-phase application remained unexplored. In contrast, our work demonstrates for the first time that CuS achieves 66% H_2_S conversion in the gas phase under 254 nm irradiation, outperforming both TiO_2_ benchmarks and photolysis alone. This establishes the novelty of our study in advancing gas-phase photochemical H_2_S valorization, bridging fundamental plasmonic behavior with practical conversion performance.

XRD analysis confirmed single-phase CuS in the pristine catalyst, while the spent catalyst showed additional reflections attributable to elemental sulfur deposition from H_2_S decomposition (Fig. S6). XRD patterns of TiO_2_ confirmed a pure anatase phase, with no secondary phases detected after reaction. UV–vis diffuse reflectance spectra revealed a band gap of ∼2.0 eV for CuS and 3.4 eV for pristine TiO_2_, consistent with their photocatalytic activity. TiO_2_ exhibited a red shift due to sulfur interaction (see Fig. S7).

**Fig. 9:**
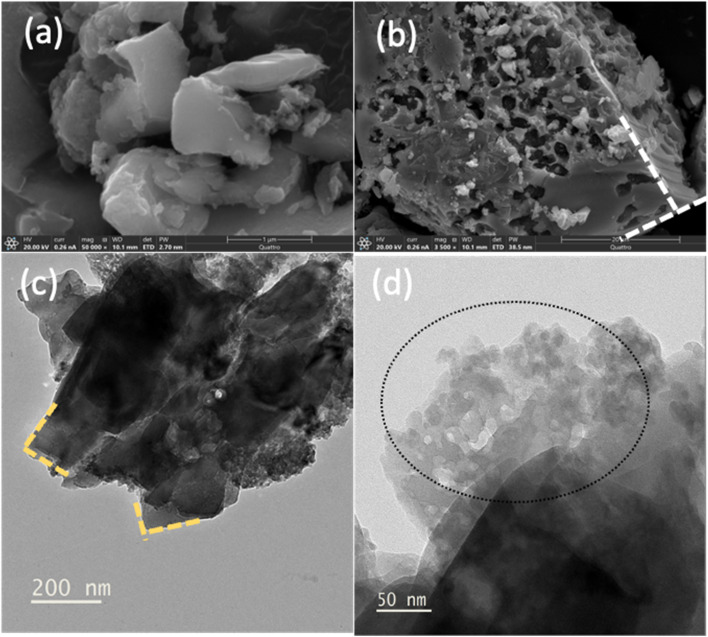
(a and b) SEM showing stacked plate-like CuS with inter-plate porosity and exposed edges. (c) TEM of an ultrathin nanosheet plate highlighting thin edge regions. (d) Higher-magnification TEM revealing nano crystallites.

Structural analysis was carried out using scanning electron microscopy (SEM) and transmission electron microscopy (TEM). SEM (Fig. 9a and b) and TEM (Fig. 9c and d) analyses show that the pristine CuS adopts a hierarchical plate-like architecture. In low-magnification SEM, micron-scale plates stack loosely, leaving inter-plate voids that form a porous network. At higher magnification, exposed edges and terraces are evident, consistent with the lamellar growth habit of covellite-type CuS. TEM further resolves ultrathin 2D nanosheets, a structural motif expected to (i) increase the accessible surface area and density of active edge sites, (ii) enhance internal light scattering within the stacked plate ensemble, and (iii) amplify local electromagnetic fields at sharp features, conditions favorable for localized surface plasmon resonance (LSPR) in p-type copper sulfides. For comparison, SEM and TEM images of TiO_2_ are provided in Fig. S8, where high-resolution TEM highlights its highly porous morphology.

**Fig. 10:**
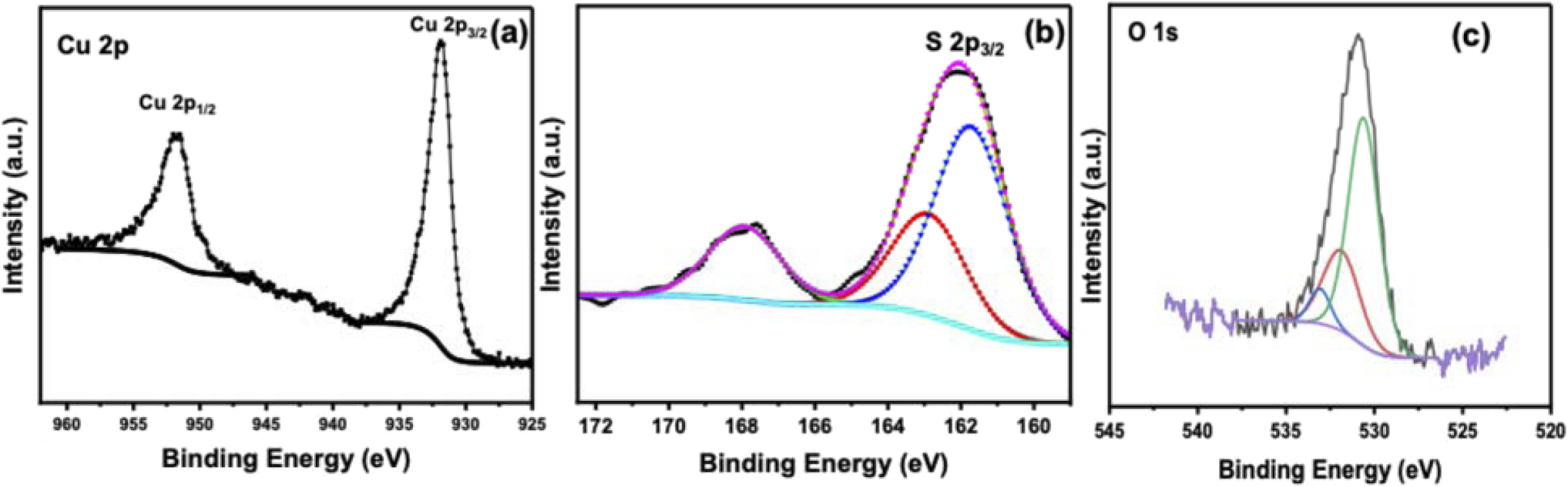
High-resolution XPS spectra of CuS: (a) Cu 2p region showing Cu valence states, (b) S 2p region confirming S^2−^ and surface S^0^ species, and (c) O 1s region indicating adsorbed hydroxyl groups and minor surface oxygen species.

The surface chemical states of the synthesized CuS catalyst were investigated by X-ray photoelectron spectroscopy (XPS) to elucidate the nature of active species responsible for photocatalytic H_2_S decomposition. High-resolution spectra of Cu 2p, S 2p, and O 1s regions were recorded and deconvoluted to distinguish contributions from different oxidation states and surface species. The Cu 2p spectrum (Fig. 10a) displays two principal peaks at 931.7 eV (Cu 2p_3_/_2_) and 951.5 eV (Cu 2p_1_/_2_), assigned predominantly to Cu^+^ species. Notably, the absence of shake-up satellite features in the 940–945 eV region, thereby confirming the covellite-type CuS phase as the dominant surface state.^42,43^ Quantitative analysis shows the S 2p_3/2_ peak (Fig. 10b) at 161.7 eV (53.91% area) and a secondary S 2p_1_/_2_ at 162.8 eV (29.96% area), indicative of sulfide ions (S^2−^) and minor surface-bound sulfur (S^0^), respectively. The SO_4_^2−^ signal at 167.9 eV (19.13%) points to trace oxidation, likely due to ambient exposure. The absence of pronounced Cu^2+^ satellite features in the Cu 2p region further substantiates the dominance of Cu^+^ and confirms minimal surface oxidation. This electronic structure supports a robust LSPR (localized surface plasmon resonance) response, arising from the mixed-valence states and copper vacancies endemic to covellite, and thus facilitates effective charge separation and high photocatalytic activity under UV illumination.^44^

The O 1 s spectrum (Fig. 10c) displays a weak peak at ∼531.5–532.5 eV, corresponding to adsorbed hydroxyl groups (OH^−^) or molecularly physiosorbed water.^45^ The absence of strong lattice oxygen signals indicates minimal bulk oxidation of CuS. Surface hydroxylation may enhance H_2_S adsorption and facilitate proton-coupled electron transfer reactions without compromising the structural integrity of the catalyst.

The XPS spectra of spent CuS catalyst exhibits distinct changes in its XPS spectra (Fig. S9), signifying surface and compositional transformations. The primary Cu 2p_3_/_2_ peak shifts to 932.8 eV and Cu 2p_1_/_2_ to 952.7 eV, reflecting mild oxidation or an increase in copper valency near the surface as a consequence of catalytic cycling. The S 2p_3_/_2_ region also shifts upward to 162.7 eV (57.9% area), with the S 2p_1_/_2_ peak at 163.9 eV (28.7% area), indicating a higher fraction of intermediate sulfur states. These binding energies are consistent with elemental sulfur (S^0^) or polysulfide (S_*n*_^2−^) species, which are known to form during photocatalytic H_2_S decomposition. The contribution of sulfate (SO_4_^2−^, 168.8 eV) decreases to 13%, suggesting that surface species were partially consumed or removed during reaction, likely *via* transformation into lower-valence sulfur species or desorption under operating conditions. Such shifts in Cu and S binding energies are emblematic of dynamic surface processes that accompany robust photocatalytic activity. Notably, the persistence of a mixed-valence environment supports continued LSPR capability, though changes in surface composition may gradually impact efficiency and long-term stability.

**Fig. 11:**
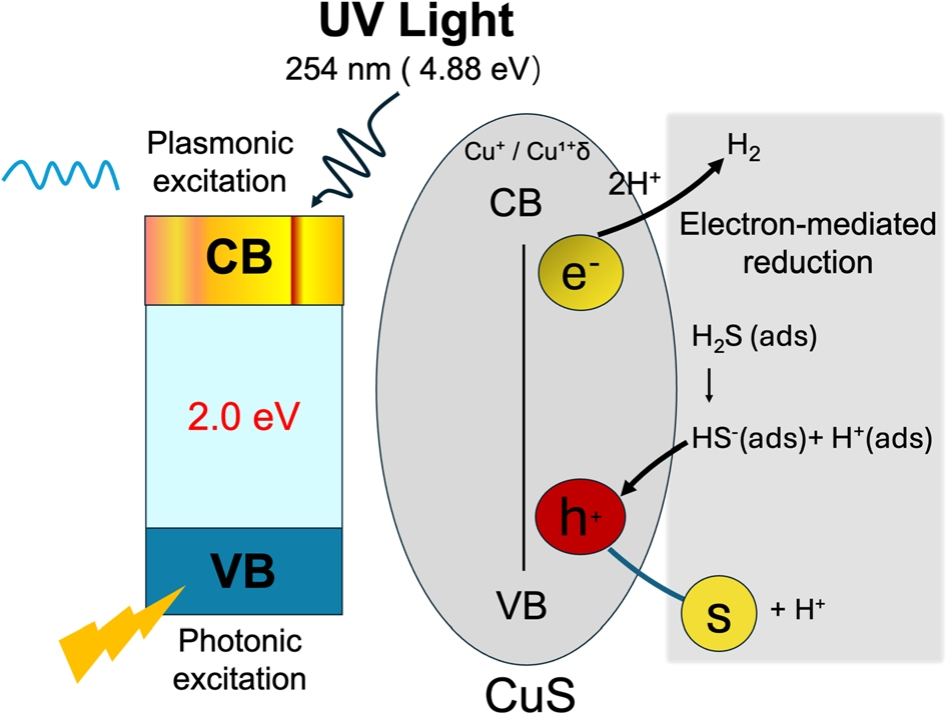
Schematic illustration of the photocatalytic and plasmon-assisted decomposition of H_2_S over CuS under UV illumination.

Following the XPS characterization, a mechanistic model is proposed to correlate the surface chemical states of CuS with its photocatalytic performance under 254 nm illumination (Fig. 11). The UV photons (4.88 eV) possess energy greater than the CuS band gap (∼2.1 eV), enabling efficient interband excitation of electrons from valence band (VB) to conduction band (CB). The XPS results confirms that Cu in CuS is predominantly in +1 oxidation state, with minor contributions from non-stoichiometric delocalized Cu^1+^δ surface species consistent with copper deficit covellite type CuS. Such copper vacancies generate high hole densities that know to support plasmon resonance (LSPR), which further enhances charge carrier generation and separation.^46,47^ Valence band holes (h^+^) oxidize the adsorbed H_2_S molecules to protons (H^+^) and elemental sulfur (S^0^), while CB electrons and plasmonic hot electrons reduce the liberated protons to molecular hydrogen (H_2_). Surface hydroxyl groups and sulfur species identified by XPS are likely to facilitate adsorption and proton-coupled electron transfer. Thus, the interplay of interband excitation and vacancy-induced plasmonic activity provides a synergistic pathway for efficient H_2_S decomposition into H_2_ and S^0^.

## 4. Conclusions

This study systematically investigated the photolytic decomposition of H_2_S using two UV sources (a UV laser at 220 nm and a Hg lamp at 254 nm), thereby illuminating different reactor systems. Under UV irradiation at wavelengths of 220 nm and 254 nm, remarkable efficiency in H_2_S splitting, yielding nearly equimolar hydrogen in the gas phase, was demonstrated. At 220 nm, conversion efficiency of ∼8.54 × 10^−7^ of H_2_S per joule was achieved. Meanwhile, photolysis experiments at 254 nm yielded conversion efficiency of ∼4.4 × 10^−9^ moles of H_2_S per joule which demonstrated the significantly higher efficiency of 220 nm wavelength in dissociating H–S bond in H_2_S molecules. In flow experiments, at a GHSV = 15 h^−1^ of 15 h^−1^ with a high concentration (5% H_2_S/N_2_), we achieved ∼13–16% conversion. The sulfur powder produced during the reaction presents a significant challenge for utilizing the full potential of UV light. However, heating and proper quenching of sulfur can provide a viable solution for photochemical H_2_S removal technology.

Incorporating CuS significantly enhanced photocatalytic activity, achieving ∼66% H_2_S conversion within 60 minutes under 254 nm UV illumination, outperforming photolysis alone (∼52%). XPS analysis revealed that copper is predominantly present as Cu^+1^, with higher binding energy contributions attributed to partially oxidized or delocalized Cu^1+^δ states. This electronic structure enables localized surface plasmon resonance (LSPR) and stabilizes hot carriers, thereby improving charge separation and reactivity. S 2p spectra further confirmed the formation of elemental sulfur (S^0^) during the reaction, while XRD of spent catalysts identified crystalline sulfur phases without significant CuS degradation, highlighting structural resilience.

Collectively, these findings highlight the potential of CuS-based photocatalytic systems for sustainable hydrogen and sulfur production from H_2_S. Addressing sulfur deposition effects and optimizing reactor configurations will be critical for translating this approach into practical, scalable applications. Future research should aim to deepen mechanistic understanding and refine catalyst design to maximize efficiency and stability in real-world conditions.

## Conflicts of interest

There are no conflicts to declare.

## Supplementary Material

RA-015-D5RA04250J-s001

RA-015-D5RA04250J-s002

## Data Availability

The data supporting the findings of this study, including raw gas chromatographic measurements, XPS spectra, and optical power measurements, are available from the corresponding author upon request. Supplementary information: Additional supporting figures and videos are provided in the Supplementary Information (SI). See DOI: https://doi.org/10.1039/d5ra04250j.
