# Supplementary material for: Photochemical valorization of hydrogen sulfide: a study of UV-induced decomposition pathways
Source: RSC Adv. 2025 Oct 7;15(44):37299–308. doi: 10.1039/d5ra04250j (PMC12501960; doi:10.1039/d5ra04250j)
Supplement: RA-015-D5RA04250J-s001 [file RA-015-D5RA04250J-s001.pdf]

Supplementary Information (SI) for RSC Advances.
This journal is © The Royal Society of Chemistry 2025

Supplementary information

Photochemical Valorization of Hydrogen Sulfide: A Study of UV-Induced Decomposition Pathways

Hassnain Abbas Khan[a], Ali Elkhazraji [b,c], Mohammad Abou-Daher[a], Damian P San Roman Alerigi[d], Khalid Hazazi[d], Aamir Farooq[a*]

[a]Clean Energy Research Platform, Physical Sciences and Engineering Division, King Abdullah University of Science and Technology (KAUST), Thuwal 23955-6900, Kingdom of Saudi Arabia

[b]Department of Mechanical Engineering, King Fahd University of Petroleum & Minerals, Dhahran 31261, Saudi Arabia.

[c]Interdisciplinary Research Center for Hydrogen Technologies and Carbon Management, King Fahd University of Petroleum & Minerals, Dhahran 31261, Saudi Arabia.

[d]Exploration and Petroleum Engineering Center-Advanced Research Center (EXPEC ARC), Saudi Aramco, Dhahran 34465, Saudi Arabia

**Corresponding author: Aamir Farooq (aamir.farooq@kaust.edu.sa)*

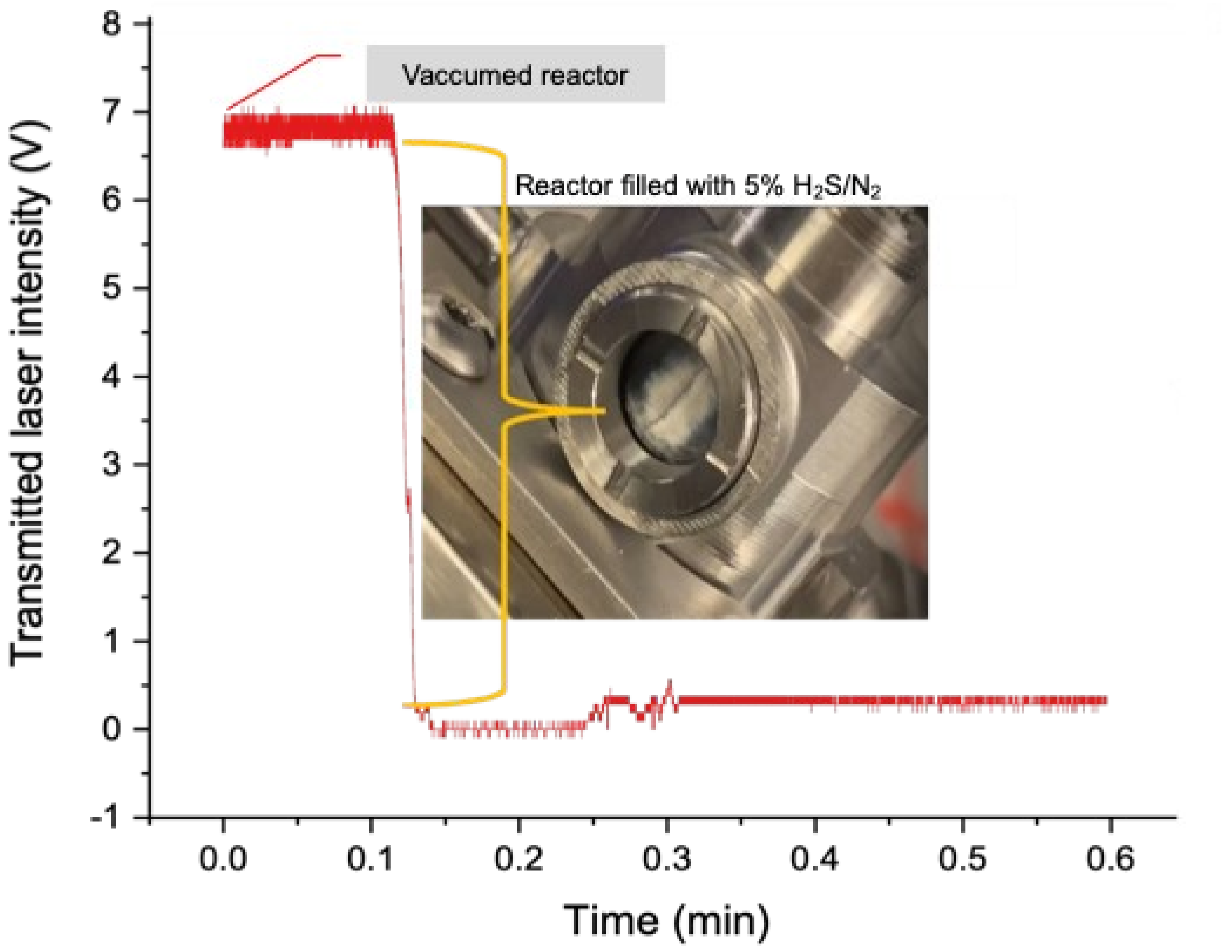

Figure S1. Transmission of laser light using photodetector through the optical windows

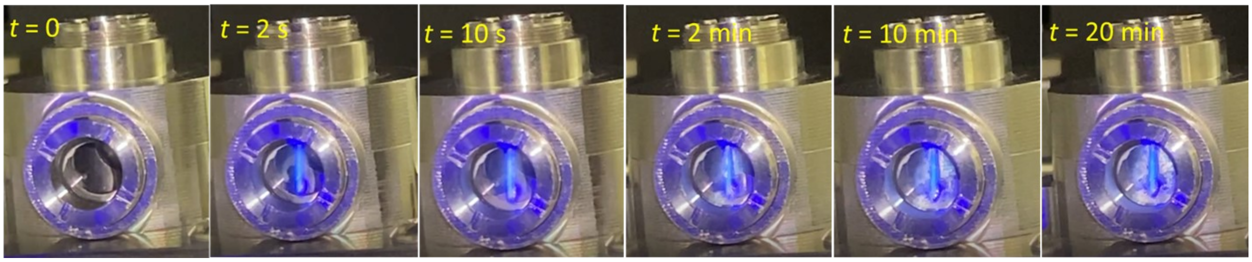

Figure S2. UV irradiation and time laps of 10% H_2S/N_2 decomposition: Sulfur formation on optical windows at optical power of 25 mW

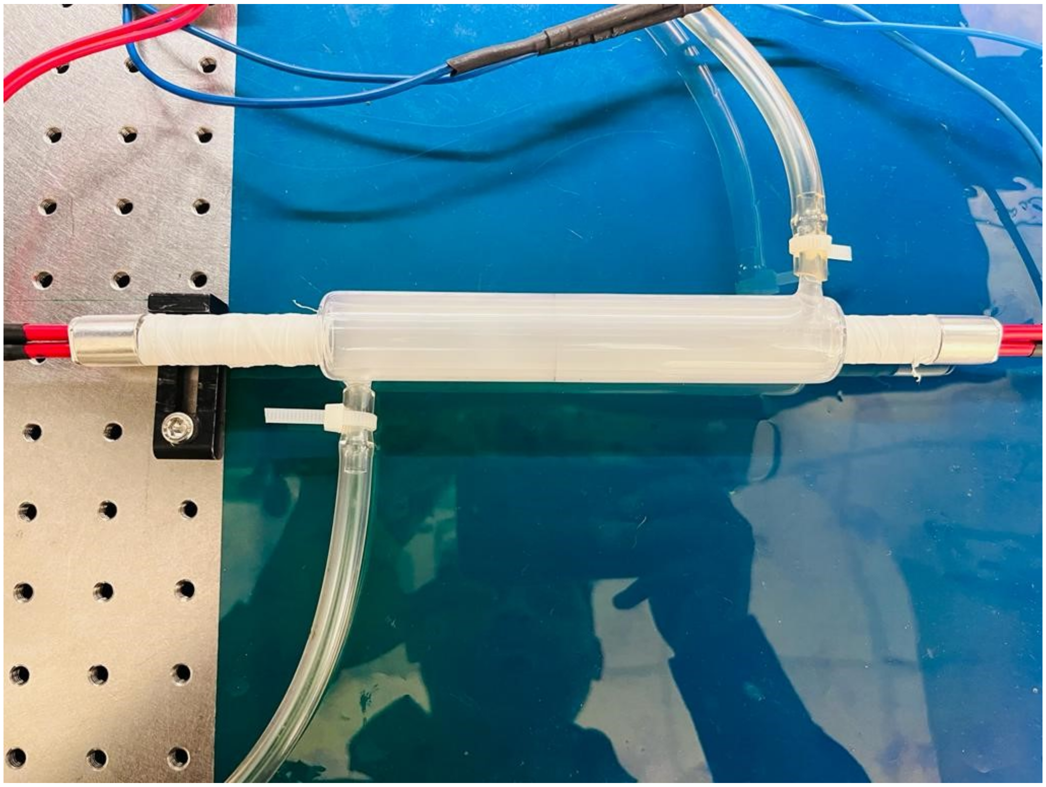

Figure S3. Image taken after 3-hour experiment with 10% H_2S/N_2 under UV lamp illumination at 254 nm.

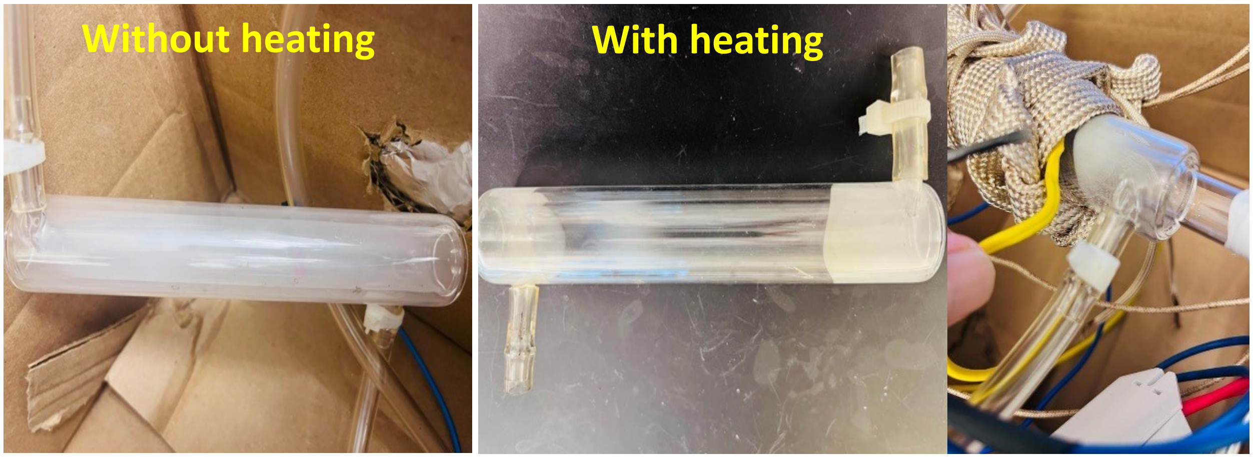

Figure S4. Image taken after 3-hour experiment with 10% H_2S/N_2 under UV lamp illumination at 254 nm with and without heating to 125 ^{o}C

1. Catalyst synthesis for photocatalytic reaction

1.1 Synthesis of CuS catalyst

The catalyst synthesis method was adapted from the literature [1] with slight modifications, as detailed in Figure S5. Briefly, 50 mL of deionized (DI) water was taken in a beaker, and 0.025 mol of CuCl was added, followed by stirring (pH = 6). To this solution, 1 mL of triethanolamine (TEA) and 2 mL of isopropyl alcohol (IPA) were added, and the mixture was stirred vigorously for 30 minutes at 70°C (pH = 8). This mixture was labeled as the Cu-precursor solution. Next, 0.053 mol of thiourea (CH_4N_2S) was directly added to the Cu-precursor solution. The total volume of the solution was then brought up to 200 mL by adding DI water, and the pH was adjusted to ~10 using NaOH solution. The mixture was allowed to react for 2 hours at a constant temperature of 70°C. After the reaction, the solution was cooled naturally to room temperature. A greenish-black precipitate was obtained, which was collected by centrifugation at 3200 RPM for 10 minutes following successive washing steps with DI water and ethanol three times to remove by-products and impurities. Finally, the product was dried in an oven at 100°C for 2 hours in an open atmosphere.

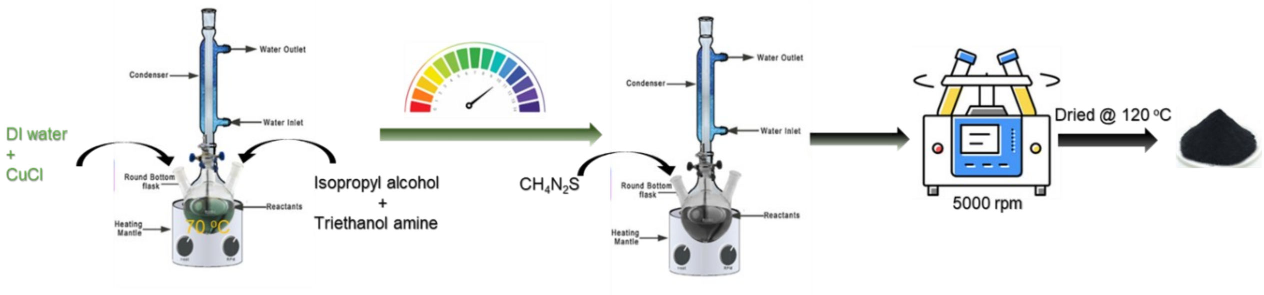

Figure S5. Experimental schematic for the synthesis of CuS photocatalyst.

Characterization Methods

The crystal structure of the catalyst was characterized by powder X-ray diffraction (XRD) on a Bruker D8 Advance diffractometer, using Cu Kα radiation (λ = 1.5418 Å) operated at 40 kV and 40 mA, over a 2θ range of 10–90°.

The morphology of the optimized catalyst for dry methane combustion was examined by scanning transmission electron microscopy (STEM) combined with energy-dispersive X-ray spectroscopy (EDX), performed on a Thermo Fisher Scientific Titan Cs-Probe microscope equipped with a Schottky field emission gun and operated at an accelerating voltage of 300 kV.

Surface compositions and chemical states were analyzed by X-ray photoelectron spectroscopy (XPS) using a Kratos Amicus instrument. High-resolution XPS (HrXPS) measurements were further carried out on a Kratos Axis Ultra spectrometer (Kratos Analytical Ltd.) equipped with a monochromatic Al Kα X-ray source (hv = 1486.6 eV, 150 W). The spectrometer, operated under a vacuum of 1×10^{-8} mbar, was fitted with a multichannel plate and delay line detector. Prior to analysis, samples were etched with an Ar^+ ion beam for 30 min. Survey spectra and high-resolution spectra were recorded at pass energies of 160 eV and 20 eV, respectively. Binding energies were calibrated against the C 1s reference peak at 284.8 eV. Data processing and deconvolution of the spectra were performed using the CasaXPS software package. UV–Vis diffuse reflectance spectra were recorded on a PerkinElmer Lambda 5000 UV–Vis spectrometer to investigate the optical absorption properties of the catalyst.

X-ray Diffraction analysis

The pristine CuS powder catalyst exhibited diffraction peaks characteristic of single-phase CuS at 2θ values of 27.69°, 29.25°, 31.93°, 47.98°, and 58.66°. In contrast, the spent catalyst showed additional reflections at 27.69° (100), 29.25° (101), 31.80° (102), 38.82° (006), 47.90° (105), and 58.68° (108). Notably, new peaks emerged at 23.11° and 25.89° after 254 nm UV lamp exposure under reaction conditions (5% H_2S/N_2). These peaks overlap with sulfur signatures and are attributed to physiosorbed elemental sulfur formed during H_2S decomposition. The reflections at 23.11° and 25.89° match well with orthorhombic α-sulfur (S_8), in agreement with JCPDS 00-013-0148. In comparison, TiO_2 samples (Figure S6) exhibited a pure anatase phase, with no secondary phases detected.

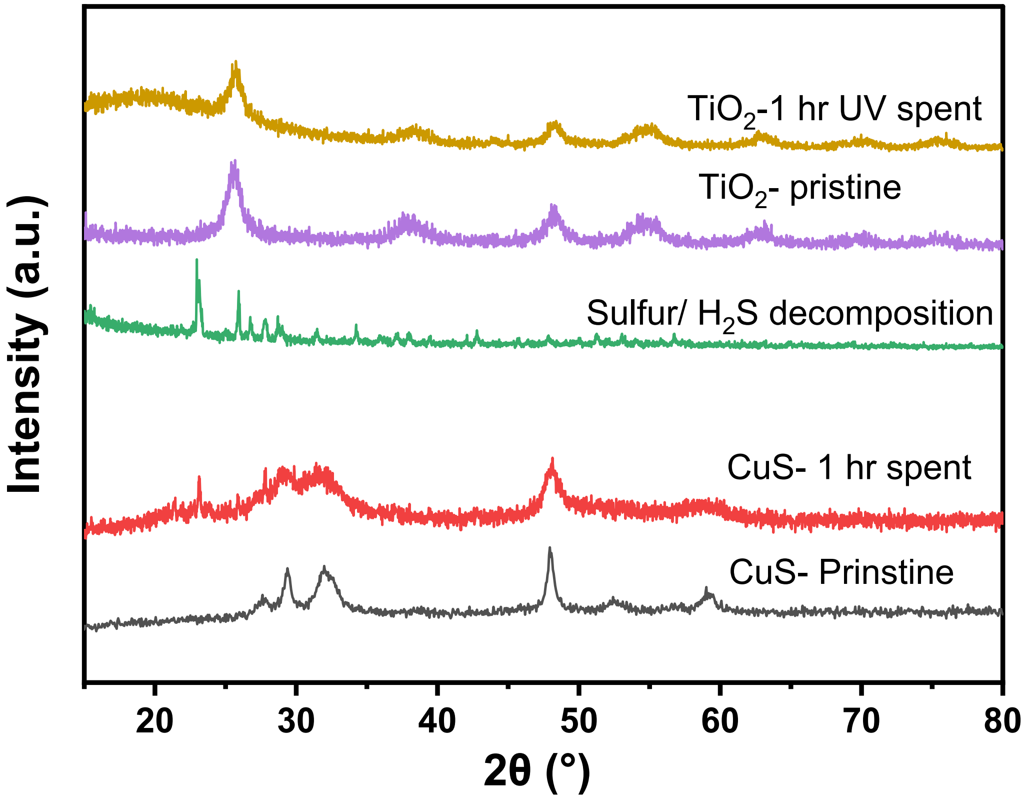

Figure S6. X-ray diffraction patterns of pristine and spent CuS and TiO_2 photocatalyst.

1.2 UV-vis diffuses reflectance spectra of CuS and TiO_2 catalyst

The UV-vis diffuse reflectance spectra of CuS and TiO_2 catalysts were measured over the wavelength range of 200–1000 nm, as shown in Figure S7. The CuS catalyst exhibited broad absorption from 250 to 400 nm, with distinct peaks at 270 nm and 385 nm, and a gradual increase in absorption beyond 600 nm into the infrared region. The tailing in the infrared range is attributed to interband transitions, while the band gap energy, calculated to be 2.0 eV. Similarly, the TiO_2 catalyst demonstrated strong UV absorption, characteristic of its anatase phase, with electron transitions from the valence band to the conduction band. The fresh TiO_2 has direct band gap of 3.4 eV. In the spent TiO_2 sample, a red shift in absorption was observed. The plausible reason for this shift is attributed to the sulfur species reacting with TiO_2, as evidenced by the color change of TiO_2 from white to yellowish after the reaction. This interaction led to partial catalyst corrosion, reducing the photocatalytic performance.

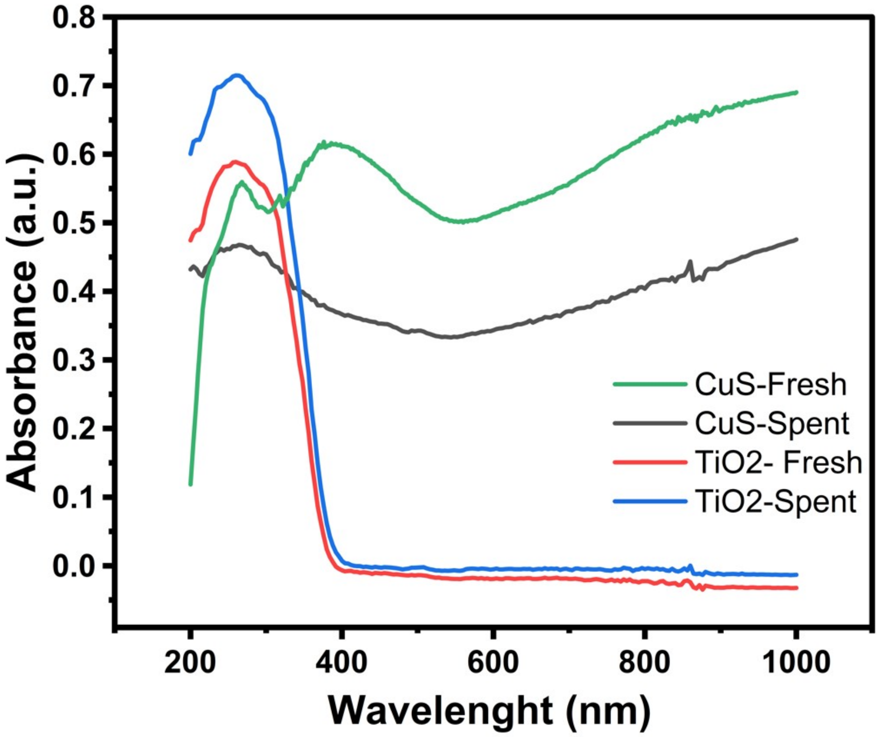

Figure S7. UV-vis spectra of fresh and spent CuS and TiO_2-G5 catalysts, illustrating changes in optical properties before and after the reaction.

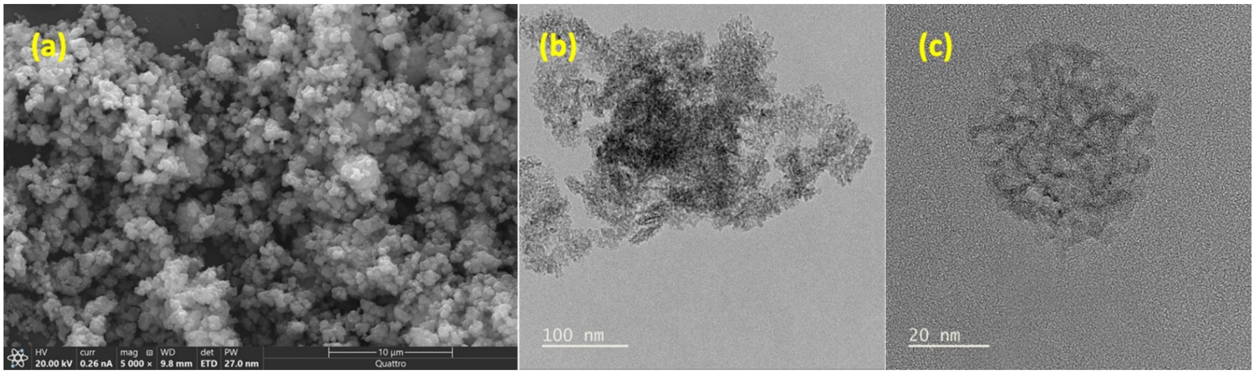

Figure S8. (a) SEM image of TiO_2 (b–c) TEM images of TiO_2 Photocatalyst

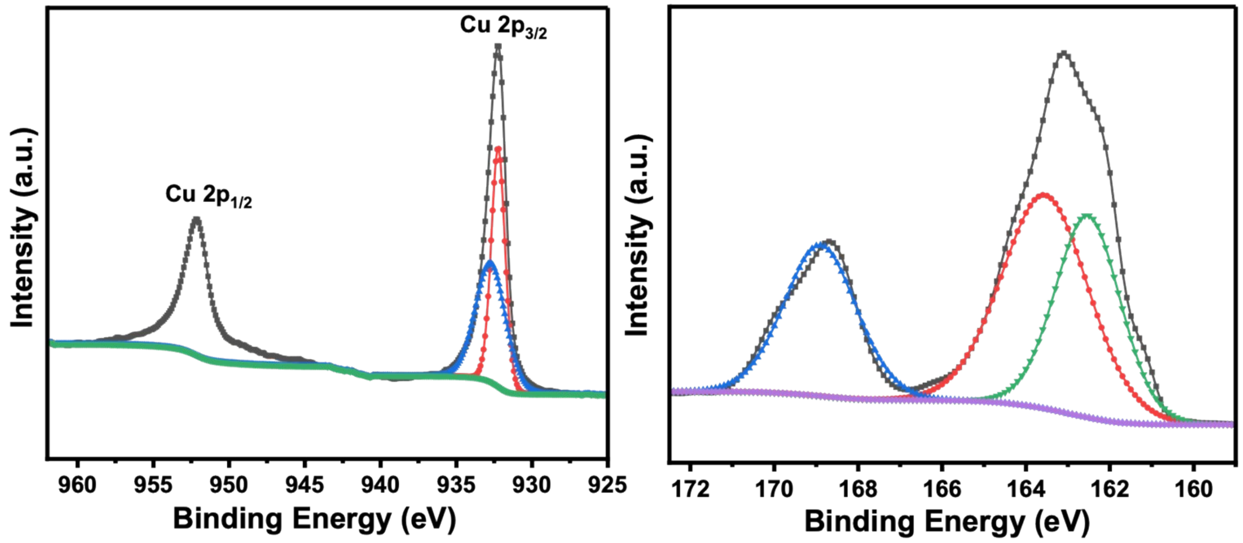

Figure S9. High-resolution XPS spectra of CuS: (a) Cu 2p region showing Cu valence states, (b) S 2p region confirming sulfur oxidation states in spent (at UV- 254 nm) catalyst

Table S1. Benchmarking H_2S Conversion Performance Against Literature

Study	λ (nm)	Catalyst	Conversion Efficiency (%)	Key Findings
Baldovi et al. (2017) [Batch]	254	Photolysis	40	Achieved stoichiometric H_2 and S production in gas phase without catalysts.
Uesugi et al. (2022) [Batch]	254	TiO_2 nanotubes	52	High degradation rates using anatase TiO_2 nanotubes in gas phase.
Dan et al. (2020) [Batch]	>420	CuS	58	Highlighted CuS stability and efficiency under visible light in aqueous phase.
Vikrant et al. (2019) [Batch]	254	TiO_2	30	Lower efficiency due to sulfur poisoning; benchmark for non-plasmonic catalysts.
Current Study [Batch]	254	CuS	66	Plasmonic CuS enhanced charge separation, outperforming pure photolysis.
Current Study [Batch]	220	Photolysis	51	Achieved robust conversions as documented by real time video and efficiency is higher than higher wavelengths (i.e 254 nm)
Current Study [Flow]	220	Photolysis	15	Demonstrated feasibility in continuous flow.

Reference:

[1] B. Pejjai, M. Reddivari, T.R.R. Kotte, Phase controllable synthesis of CuS nanoparticles by chemical co-precipitation method: Effect of copper precursors on the properties of CuS, Mater Chem Phys 239 (2020). https://doi.org/10.1016/j.matchemphys.2019.122030.
